# Optimizing the Yield of Multi-Unit Activity by Including the Entire Spiking Activity

**DOI:** 10.3389/fnins.2019.00083

**Published:** 2019-02-12

**Authors:** Eric Drebitz, Bastian Schledde, Andreas K. Kreiter, Detlef Wegener

**Affiliations:** Brain Research Institute, Center for Cognitive Science, University of Bremen, Bremen, Germany

**Keywords:** monkey, primary visual cortex, extracellular recordings, receptive field mapping, signal-to-noise ratio, multielectrode arrays, semi-chronic recordings

## Abstract

Neurophysiological data acquisition using multi-electrode arrays and/or (semi-) chronic recordings frequently has to deal with low signal-to-noise ratio (SNR) of neuronal responses and potential failure of detecting evoked responses within random background fluctuations. Conventional methods to extract action potentials (spikes) from background noise often apply thresholds to the recorded signal, usually allowing reliable detection of spikes when data exhibit a good SNR, but often failing when SNR is poor. We here investigate a threshold-independent, fast, and automated procedure for analysis of low SNR data, based on fullwave-rectification and low-pass filtering the signal as a measure of the entire spiking activity (ESA). We investigate the sensitivity and reliability of the ESA-signal for detecting evoked responses by applying an automated receptive field (RF) mapping procedure to semi-chronically recorded data from primary visual cortex (V1) of five macaque monkeys. For recording sites with low SNR, the usage of ESA improved the detection rate of RFs by a factor of 2.5 in comparison to MUA-based detection. For recording sites with medium and high SNR, ESA delivered 30% more RFs than MUA. This significantly higher yield of ESA-based RF-detection still hold true when using an iterative procedure for determining the optimal spike threshold for each MUA individually. Moreover, selectivity measures for ESA-based RFs were quite compatible with MUA-based RFs. Regarding RF size, ESA delivered larger RFs than thresholded MUA, but size difference was consistent over all SNR fractions. Regarding orientation selectivity, ESA delivered more sites with significant orientation-dependent responses but with somewhat lower orientation indexes than MUA. However, preferred orientations were similar for both signal types. The results suggest that ESA is a powerful signal for applications requiring automated, fast, and reliable response detection, as e.g., brain-computer interfaces and neuroprosthetics, due to its high sensitivity and its independence from user-dependent intervention. Because the full information of the spiking activity is preserved, ESA also constitutes a valuable alternative for offline analysis of data with limited SNR.

## Introduction

As an early step during analysis of extracellularly recorded signals, the actual spiking response of a neuron, or a group of neurons, usually needs to be separated from the background noise of the recorded signal. A common procedure is to set up an amplitude threshold to the high-passed neuronal signal. This threshold can be set manually or be defined automatically based on statistical likelihood. Frequently used methods for automatic threshold definitions use multiples of the standard deviation of the high-passed signal (Pouzat et al., 2002) or the median of the absolute signal (Quiroga et al., 2004). Amplitude threshold-based spike detection has been proven successful in data with good SNR, but its performance declines significantly with decreasing SNR (Nenadic and Burdick, 2005). Other methods, such as template matching (Bankman et al., 1993) and wavelet-based extraction of time- and frequency-resolved spike features (Yang and Shamma, 1988; Hulata et al., 2002; Quiroga et al., 2004; Nenadic and Burdick, 2005) either require a priori knowledge about the spike form, or an extensive amount of processing (Obeid and Wolf, 2004). Yet, robust methods for dealing with low SNR data become particularly important with the increased importance of multi-electrode arrays used for large-scale neuronal recordings and brain-computer interfacing (Buzsáki, 2004; Lebedev and Nicolelis, 2006; Lewis et al., 2015), and other semi-chronic recording techniques (deCharms et al., 1999; Galashan et al., 2011; Mendoza et al., 2016). In contrast to acute recordings with separately controlled microelectrodes, however, the position of array electrodes is fixed, or electrodes are more difficult to adjust. It is hence either impossible or difficult to carefully guide individual electrodes for optimizing a neuron's signal, resulting in highly variable magnitudes of extracellular action potentials (Gold et al., 2006). Additionally, signals of (semi-) chronically implanted electrodes degrade over time, due to local tissue responses (Schwartz, 2004; Polikov et al., 2005). Both issues are likely to result in a high number of channels exhibiting low SNR.

Analysis of such data is usually confined to the local field potential (LFP), because thresholding spikes in low SNR responses potentially results in a high number of either false positives or false negatives, depending on the threshold level. Hence, thresholding may have a significant impact on the estimated strength and temporal structure of the response, and interpretation of such data is problematic. The LFP, on the other hand, represents the integrated neuronal activity in close neighborhood of the electrode and constitutes a sensitive measure of neuronal activity (Liu and Newsome, 2006; Katzner et al., 2009). Yet, the LFP reflects the sum of all local transmembrane currents rather than the output signal of the recorded neurons. Analysis of the latter, therefore, requires a reliable method to efficiently segregate stimulus responses from unspecific background noise, particularly at low and medium SNR recording sites. At the same time, there should be no trade-off at recording sites with high SNR when compared to established methods based on thresholding.

We hypothesized that a method introduced in the early 1990s by Eckhorn and colleagues (Eckhorn, 1991, 1992; Eckhorn and Obermüller, 1993; Brosch et al., 1997) possesses the critical properties to serve as such a reliable signal for detecting evoked responses in low SNR data. This method was invented for analyzing correlated activity at multi-unit recording sites, and is based on a fullwave-rectification of the high-passed neuronal signal (containing the spike information), followed by low-pass filtering. The method delivers a continuous instead of a binary signal, and represents the aggregated spiking activity of neurons located about 50 μm around the electrode's tip (Legatt et al., 1980; Brosch et al., 1997). Its most important advantage is that it does not rely on setting up a threshold but takes all the available spiking information. Because of the final low-pass filtering it should be rather insensitive to random high-frequency noise, making it a highly promising candidate approach for detecting evoked responses when SNR is weak. For the remainder of the paper, we denote this signal as ESA (Entire Spiking Activity).

Since its introduction, ESA has been used as an alternative measure for multi-unit activity by several groups (Self et al., 2016; Dougherty et al., 2017; Drebitz et al., 2018), but many of its important properties are still awaiting quantitative description. The purpose of the present study is to analyze the potential of ESA for increasing the yield of multi-unit recordings at different SNRs, and to quantitatively compare evoked responses based on ESA and thresholded MUA. For the example of receptive field (RF) mapping, we analyze semi-chronic recordings from primary visual cortex (V1) of five macaque monkeys (*Macacca mulatta*), and compare ESA-based RF detection rates with both conventionally thresholded MUA and the LFP, and further analyze RF size and orientation selectivity between ESA- and MUA-based RFs obtained from the same high-frequency signal. We use two approaches to set the threshold for analyzing MUA: a standard procedure with a fixed threshold for all units, and a second, computationally time-consuming iterative procedure to determine the optimal threshold for each unit individually. The results show that ESA outperformed MUA in both cases, particularly when SNR was low. ESA-based RF detection was almost as sensitive as LFP-based detection, and RF parameters corresponded to those found with thresholded MUA. RF-sizes were slightly larger than MUA-RFs, due to considering all available spiking information, but size differences were consistent over all SNR fractions. Relative orientation sensitivity (i.e., number of sites with significantly biased responses for different orientations) was higher for ESA, while absolute orientation selectivity (i.e., orientation indexes) was slightly attenuated as compared to thresholded MUA. Independent of these differences, the majority of recording sites delivering a RF with both signal types was found to have similar preferred orientations. Thus, ESA constitutes a powerful source of information to be considered when depending on reliable and fast neuronal response detections, such as for (semi-) chronic recordings or BCI-approaches, as well as for increasing the information content of low SNR data for offline analysis.

## Materials and Methods

### Subjects and Surgical Procedures

Five male macaque monkeys (*Macaca mulatta)* were implanted with custom-made head holders and recording chambers under aseptic conditions and propofol/remifentanyl anesthesia. Four animals (monkeys B, P, V, and F) were implanted with a V1 microdrive array, allowing for bidirectional movement of six semi-chronically inserted electrodes (Galashan et al., 2011). The fifth animal (monkey T) was implanted with a recording chamber located above areas V4 and V1, allowing for bidirectional movement of up to four electrodes. Details on anesthesia, analgesia, and surgical procedures are reported elsewhere (Wegener et al., 2004; Galashan et al., 2011; Schledde et al., 2017; Drebitz et al., 2018). All procedures were in accordance with the Regulations for the Welfare of Experimental Animals issued by the Federal Government of Germany and with the guidelines of the European Union (2010/63/EU) for care and use of laboratory animals, and were approved by the local authorities (Senator für Gesundheit, Bremen, Germany).

### Visual Stimuli and Behavioral Task

Data was acquired with an automatic bar-mapping procedure to stimulate the visual field region of interest, similar to the method described by Fiorani et al. (2014). The mapping was performed for different scientific projects not reported here. For the stimulation details that follow, task parameters of monkey T are stated in the text, and deviating parameters of one or more other animals are given in brackets. Visual stimulation was performed on a 20-inch (22-inch) CRT-screen, with a resolution of 1,024 × 768 (1,280 × 1,024) pixels at 100 Hz vertical refresh rate. Monkeys were placed in a custom-made primate chair 90 (80) cm in front of the screen. Appearance of the central fixation point (FP) indicated trial start and animals were given 2 s to initiate the trial by gazing at the FP and pressing a lever. Following a blank period of 820 (300) ms, a high-contrast bar appeared on screen and moved with constant speed in one of 12 motion directions (separated by 30°), and disappeared at the end of the trajectory. Length of bars (3.2–8.2°), motion trajectories (2.5–10.75°), and stimulus speed (1.9–4.7°/s) varied between animals, recording sites, and occasionally between recording sessions, depending on the spatial area to be covered (16–64 deg^2^). Monkeys were required to keep fixation throughout the trial and to indicate a decrease in FP luminance occurring during a pseudo-random interval between 250 and 1,250 ms after bar disappearance, by releasing the lever within a time period from 150 to 750 ms after FP dimming. To ensure that animals stayed alert throughout the trial, FP dimming occurred already during bar presentation in about 10% of trials. These trials did not enter data analysis. Successive trials were separated by a 2 s inter-trial interval. Eye position was monitored by video-oculography (monkey T: ISCAN Inc., MA, USA; monkeys B, P, V, and F: custom-made eye tracking system). Correctly performed trials were rewarded with a small amount of water or diluted grape juice. Responding too soon or too late, and eye movements of more than 0.5° (1°) away from the FP caused immediate trial termination without reward.

### Data Acquisition

Neuronal data was recorded using up to six epoxy- or glass-insulated tungsten electrodes (125 μm diameter, 1–3 MΩ, FHC Inc., Bowdoin, ME, USA). Two different recording setups were used for data acquisition. In the first setup (monkeys B and P), the electrode signal was sampled at 25 kHz frequency, amplified 3,000-fold (10 ×, custom-made head stage, 300 ×, custom-made main-amplifier), and band-passed between 0.7 and 5 kHz for receiving the spike information. For the LFP, the amplified electrode signal was low-passed at 300 Hz and down-sampled to 1 kHz. Hardware-filtered data was then digitized at 16 bit ADC resolution. In the second setup, the electrode signal was amplified using either a custom-made head stage (monkeys V and F), or a wideband preamplifier (monkey T; MPA32I, Multi Channel Systems, Reutlingen, Germany), both with a gain of 10, and a main-amplifier (PGA 64, 1–5,000 Hz, Multi Channel Systems, Reutlingen, Germany) with a gain of 1,000. The amplified raw-signal was digitized with a sampling-rate of 25 kHz and a resolution of 12 (monkey T) or 16 bits (monkeys V and F). Electrode signals were referenced either against a low impedance electrode (<0.1 MΩ) implanted into the frontal skull bone and touching the dura (monkeys B, P, V, F), or against the titanium recording chamber (monkey T), which was screwed into the bone and touching the dura.

### Data Analysis

All offline analyses were performed with customized MATLAB-scripts (Mathworks, Natick, MA, USA). As described above, data of monkeys B and P was already band-pass filtered before digitizing. Data of monkeys T, V, and F was filtered offline either between 0.7 and 5 kHz (monkeys V and F) or 0.3–12.5 kHz (monkey T) for isolating the high-frequency components (spikes), and low-passed either below 300 Hz (monkeys V and F) or 170 Hz (monkey T) for the low-frequency components (LFP). All offline filters were equiripple FIR-filters, applied in forward and backward direction to avoid phase shifts.

**Figure 1:**
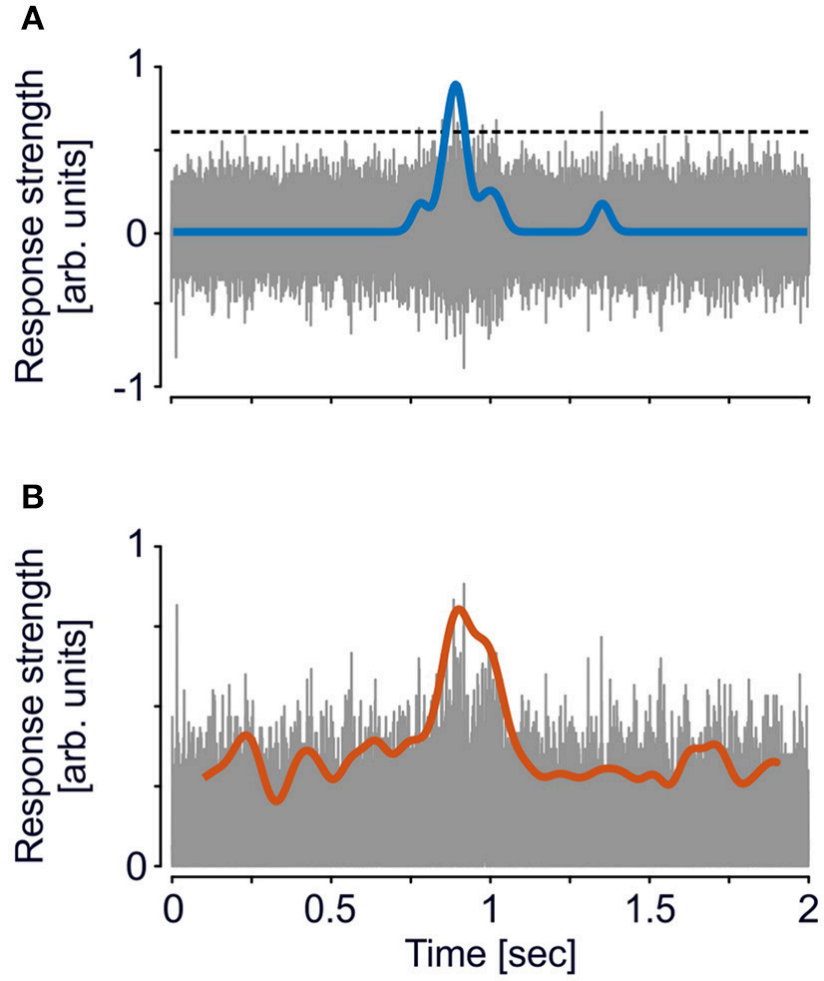
Exemplary trace of a single-trial illustrating analysis of MUA and ESA. **(A)** For MUA, a threshold (dashed line, cf. equation 1) based on the method introduced by Quiroga et al. (2004) was applied to the high-passed signal (gray trace). All events surpassing the threshold were then used for calculating the SDF (blue line). **(B)** For ESA, analysis is based on the full-wave rectified signal, i.e., the absolute values of the high-passed signal (gray trace), and then low-pass filtered (red line), using the same filter settings as for the SDF. This computation is independent of setting a threshold. Ordinate scaling is identical in both plots, SDF and ESA traces are both upscaled by a factor of 5 for visual purposes. Note that due to line thickness and time span, gaps between adjacent spike events are hardly visible. The inset in **(B)** shows a time period of 100 ms to illustrate the time course of the rectified signal in more detail.

Spike detection for analyzing thresholded MUA was done using the method introduced by Quiroga et al. (2004), defining the threshold *Thr* as:

(1) $$Thr=a*\text{median}\left(\frac{\left|\text{x}\right|}{0.6745}\right),$$

where *x* represents the high-passed data of which the median is taken and *a* represents a factor for different threshold levels. This factor was set to *a* = 3 for the standard procedure, and was varied between *a* = 2 and *a* = 4 (in steps of 0.5) for the iterative procedure. To take advantage of the full spike information, no further spike sorting was performed, and all events surpassing the threshold were used (Figure 1A). Spike times were binned with a resolution of 1 ms and convolved with a Gaussian kernel (σ = 25 ms) to obtain the spike-density function (SDF).

ESA was calculated on the same high-passed data, but instead of setting a threshold the data was full-wave rectified and low-pass filtered in forward and backward direction (Figure 1B), and down-sampled to 1 kHz (Legatt et al., 1980; Eckhorn, 1991, 1992). To achieve best comparability, low-pass filtering was performed by a Gaussian filter with the same characteristics as used for calculating the SDF.

LFP power was calculated by convolving the low-passed signal with complex Morlet's wavelets (Torrence and Compo, 1998), as described in more detail elsewhere (Tallon-Baudry et al., 1997; Taylor et al., 2005). The resulting complex coefficients $\tilde{x}$ at time *t* and frequency *f* can also be expressed by their amplitude *A* and phase Φ such that:

(2) $$\tilde{x}\left(t,f\right)=A\left(t,f\right)e^{i\Phi \left(t,f\right)}\text{.}$$

Power was calculated by taking the square of the absolute value of $\tilde{x}$(*t*,*f*), divided by the Nyquist-frequency (500 Hz). For each recording site, the power values for each time-frequency bin were normalized by first subtraction of and then division by the mean power spectrum of the spontaneous activity (obtained during the blank period prior to bar onset, excluding the first 100 ms). From this time–frequency representation of the LFP power we extracted the time course of the average power between 40 and 120 Hz.

### Receptive Field Detection

**Figure 2:**
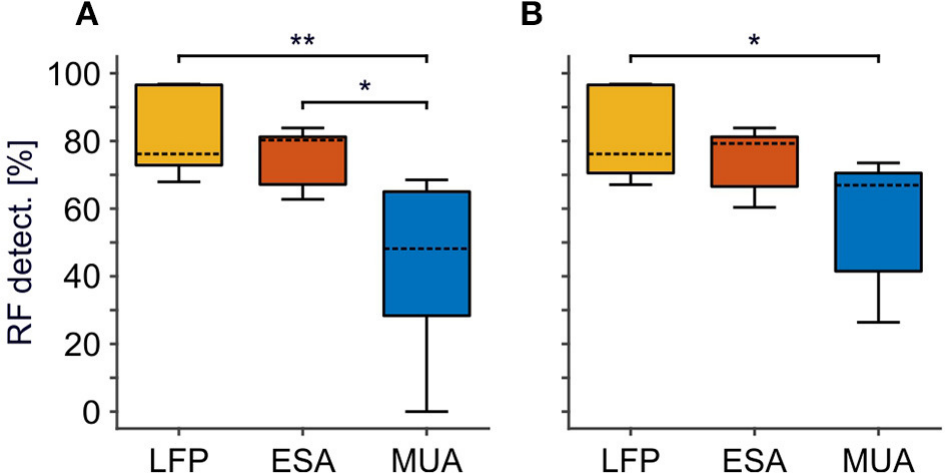
Comparison of RF detection rate between LFP, ESA, and MUA. **(A)** Standard approach for thresholding MUA. **(B)** Optimized, iterative approach for thresholding MUA to maximize MUA-based detection rate. For each signal type, boxplots are based on detection rates of individual animals. Detection rates refer to the absolute number of RFs detected relative to the number of recording sites with a significant visual activation for at least one of the signals, merged over all animals. Boxes indicates the 25th and 75th percentile, dashed lines indicate the medians, and whiskers indicate the full range of data. Asterisks indicate statistical difference for α < 0.5% (^*^) and α < 0.05% (^**^), respectively.

RF analysis was limited to data having at least five repetitions of each bar direction. To allow for direct comparison between MUA, ESA, and LFP, all data was *z*-transformed according to Fiorani et al. (2014). To this end, we first subtracted the mean spontaneous activity (averaged over all trials and orientations) from the response to a given motion trajectory, and then divided by the standard deviation of the responses to this direction. For the LFP, this was based on the average power in the broad γ-frequency range (40–120 Hz). RF-locations were calculated using the back-projection method, which is described in more detail in Fiorani et al. (2014). In brief, for each specific time point mean *z*-transformed responses to each of the 12 motion directions were back-projected to the location and orientation of the bar on screen, to obtain activity maps spanned by the bars' size and path length [see Figure 2, (Fiorani et al., 2014)]. The geometrical mean of the averaged, aligned responses to each of the 12 bar motion directions then provides the merged activity map. To avoid multiplying by values below one, each activity map with a minimum z-value < 1 was shifted by an offset parameter *R*_*off*_, given by the difference between the actual minimum value and one. The final merged map was corrected for these offsets by subtracting the geometrical *R*_*off*_ mean. We then searched for areas with mean activity higher than half the maximum of all values within the map. Such areas were considered a RF if first, the diameter (recalculated from estimated RF area) was between 0.6 and 2.6°, and second, the average *z*-value was larger than 0.8. Recording sites with low SNR sometimes contained several connected areas in their activity maps with values larger than half of the maximum amplitude. In these cases, we only considered the largest of these areas as RF, if all other areas were smaller than 0.5° in diameter. These rather conservative criteria are more likely to deliver false negatives than false positives. RF size was calculated based on the spatiotemporal resolution of the activity map and the number of entries defining the RF. With the exception of estimating significance of orientation tuning (described below), all other analyses were based on the mean *z*-transformed response within these RF borders, calculated separately for each of the 12 motion trajectories.

### Orientation Tuning

For analysis of orientation tuning, we first verified whether a site's tuning was significant (*P* < 0.05), using a method introduced by Grabska-Barwinska et al. (2012) to test the reliability of response differences to different orientations for repeated stimulus presentations. The response in any given trial is represented by the mean, non-transformed activation over the time the bar is passing the RF. In detail, for identifying significantly tuned sites, the full set of *n*_Φ_ bar orientations was shown for *n*_*k*_ repetitions, and the average in the complex plane *z*_*PO*(*k*)_ was calculated for each of the repetitions *k*:

(3) $$z_{PO\left(k\right)}=\frac{1}{n_{\Phi }}\sum_{\Phi }f\left(\Phi ,k\right)e^{2i\Phi },$$

with *f*(Φ, *k*) representing the neuronal response to each orientation Φ. The angle of *z*_*PO*(*k*)_ delivers the preferred orientation *PO* from each repetition. The vector average 〈*z*_*PO*_〉 of the normalized vectors *z*_*PO*(*k*)_ for all repetitions can then be calculated by:

(4) $$\langle z_{PO}\rangle =\frac{1}{n_{k}}\sum_{k}\frac{z_{PO\left(k\right)}}{\left|z_{PO\left(k\right)}\right|}=\frac{1}{n_{k}}\sum_{k}e^{2iPO\left(k\right)}.$$

The reproducibility of the preferred orientation *r*_*PO*_ is defined as the length of 〈*z*_*PO*_〉:

(5) $$r_{PO}=\frac{1}{n_{K}}\left|\sum_{k}e^{2iPO\left(k\right)}\right|.$$

The reproducibility is 1 if the *PO* is equal for all repetitions, and 0 if *PO* values are uniformly distributed on the 0–180° range. Significance of orientation tuning was determined by comparing the reproducibility *r*_*PO*_ to a probability distribution *P*(*r*_*PO*(_*n*__*k*_)_) obtained from Monte-Carlo simulations under the assumption of a uniform distribution of *POs*.

Sites with significant orientation tuning were used for comparing the tuning between signal types based on an orientation selectivity index calculated by vector averaging (Grinvald et al., 1986). If an average *z*-transformed response to any of the twelve bar directions was below zero, this value was multiplied with −1 and added to each of the twelve response vectors. Neuronal responses *f*(Φ) to each of the *n*_Φ_ bar directions were represented in the complex plane and averaged:

(6) $$\langle z\rangle =\frac{1}{n_{\Phi }}\sum_{\Phi }f\left(\Phi \right)e^{i2\Phi }.$$

The preferred orientation is then given by the halved angle of the average vector 〈*z*〉, and the tuning strength by its length |〈*z*〉|.

### Statistical Analysis

For each monkey and signal type, the detection ratio *DR* of significant RFs was given by:

(7) $$DR=\frac{N_{Signal\;}}{N_{all}},$$

where *N*_*Signal*_ corresponds to the number of significant RFs found for the signal type under investigation, and *N*_*all*_ corresponds to the total number of recording sites with a significant RF independent of signal type. Note that for each signal type, each recording site delivered maximally one RF by definition. Statistical analysis of detection rates was done by performing paired ANOVAs on the mean detection rates of each animal and *post-hoc* testing with Tukey's honestly significant difference (HSD) procedure, correcting for multiple comparisons. Statistical analysis of RF size and orientation selectivity was performed on sites delivering a significant RF for both ESA and thresholded MUA, pooled over all animals, using Wilcoxon signed rank tests. Effect size *R* was calculated by:

(8) $$R=\frac{\left|Z\right|}{\sqrt{N}},$$

where *Z* is taken from the Wilcoxon test statistics, and *N* represents the total number of samples.

## Results

The aim of the study was to assess the sensitivity of the fullwave-rectified, low-passed spiking activity (ESA) for unsupervised detection of visual responses. For directly comparing ESA performance under different SNR conditions with conventionally thresholded MUA and with the LFP, we used a data-set of semi-chronic intra-cortical recordings from area V1 of five macaque monkeys. Data was acquired during an automatic bar mapping procedure. We used two approaches to set the threshold for analyzing MUA-based detection. The first approach (standard procedure) used a multiplication factor of *a* = 3 for all data (see Materials and Methods). The resultant threshold level was found to be quite robust against false positives and false negatives. The second approach used an iterative procedure with multiplication factors of *a* = 2 to *a* = 4 (in steps of 0.5) to find the optimal threshold for each individual unit. Although this procedure is time-consuming and requires computing of RF maps for each threshold, it maximizes the yield of MUA-based RF detection. Note, however, that it requires a priori knowledge to distinguish evoked responses from false positives. The final dataset included all recording sites delivering an RF for at least one of the three signals types (standard procedure: *N* = 653, iterative procedure: *N* = 656).

### Quantitative Comparison of RF Detection Between Signal Types

We first analyzed RF detection rates for ESA and conventional MUA, based on signal strength and area of activation (see Materials and Methods), and compared it to LFP-based detection rates. Figure 2 provides boxplot histograms of the pooled data across signal types for each of the two MUA procedures. ESA delivered an RF at 500 recording sites, which was close to the detection rate of the LFP (*N* = 570). In contrast, MUA delivered an RF at 337 recording sites using the standard procedure, and at 399 recording sites using the iterative procedure. The latter provides the maximum of RFs to be obtained by thresholding. Table 1 summarizes the number of RFs for each individual animal and signal type. Comparing across animals, ESA-based detection delivered most RFs in three of the five animals, while in the remaining two animals most RFs were obtained using the LFP. Importantly, ESA delivered more RFs than thresholded MUA in each individual animal, regardless of the procedure to set the threshold. Note that this was true albeit individual animals were recorded for different experimental purposes, using different recording setups and filter settings, and predominant recording layers varied between animals. Thus, the higher detection rates obtained with the ESA signal were not due to specific experimental conditions but a general outcome independent of the specific recording details.

**Table 1:** Number of detected RFs for individual subjects and signal types.

**Monkey**	**Total *N***	**LFP**		**ESA**		**MUA**	
		***N***	**%**	***N***	**%**	***N***	**%**
B	130	99	76.2	109	83.9	83 (87)	63.9 (66.9)
F	81 (82)	55	67.9 (67.1)	65	80.3 (79.3)	39 (57)	48.2 (69.5)
P	219	212	96.8	176	80.4	150 (161)	68.5 (73.5)
T	172	166	96.5	118	71.1	65 (80)	37.8 (48.2)
V	51 (53)	38	74.5 (71.7)	32	62.8 (60.4)	0 (14)	0 (26.4)
Total	653 (656)	570	87.3 (86.9)	500	76.6 (76.2)	337 (399)	51.6 (60.8)

*If different between standard procedure and iterative procedure, numbers in brackets refer to the iterative procedure*.

We performed the statistical analysis on RF detection rates per thresholding procedure and across animals. For the standard procedure, a 1-way RM-ANOVA confirmed significant differences between signal-types [*F*_(2, 14)_ = 9.28, *P* = 0.008, *N* = 5]. *Post-hoc* Tukey HSD tests showed that the percentage of detected RFs based on ESA was significantly higher than detection rates for thresholded MUA (*P* = 0.026), while detection rates between ESA and LFP were statistically indistinguishable (*P* = 0.739). For the iterative procedure, the difference between ESA- and MUA-based detection now failed to reach significance (*P* = 0.113). Note, however, that also in the iterative procedure ESA delivered considerably more RFs than MUA in each individual animal (mean increase: 25%, range: 9–125%).

### Dependence of RF Detection on SNR

**Figure 3:**
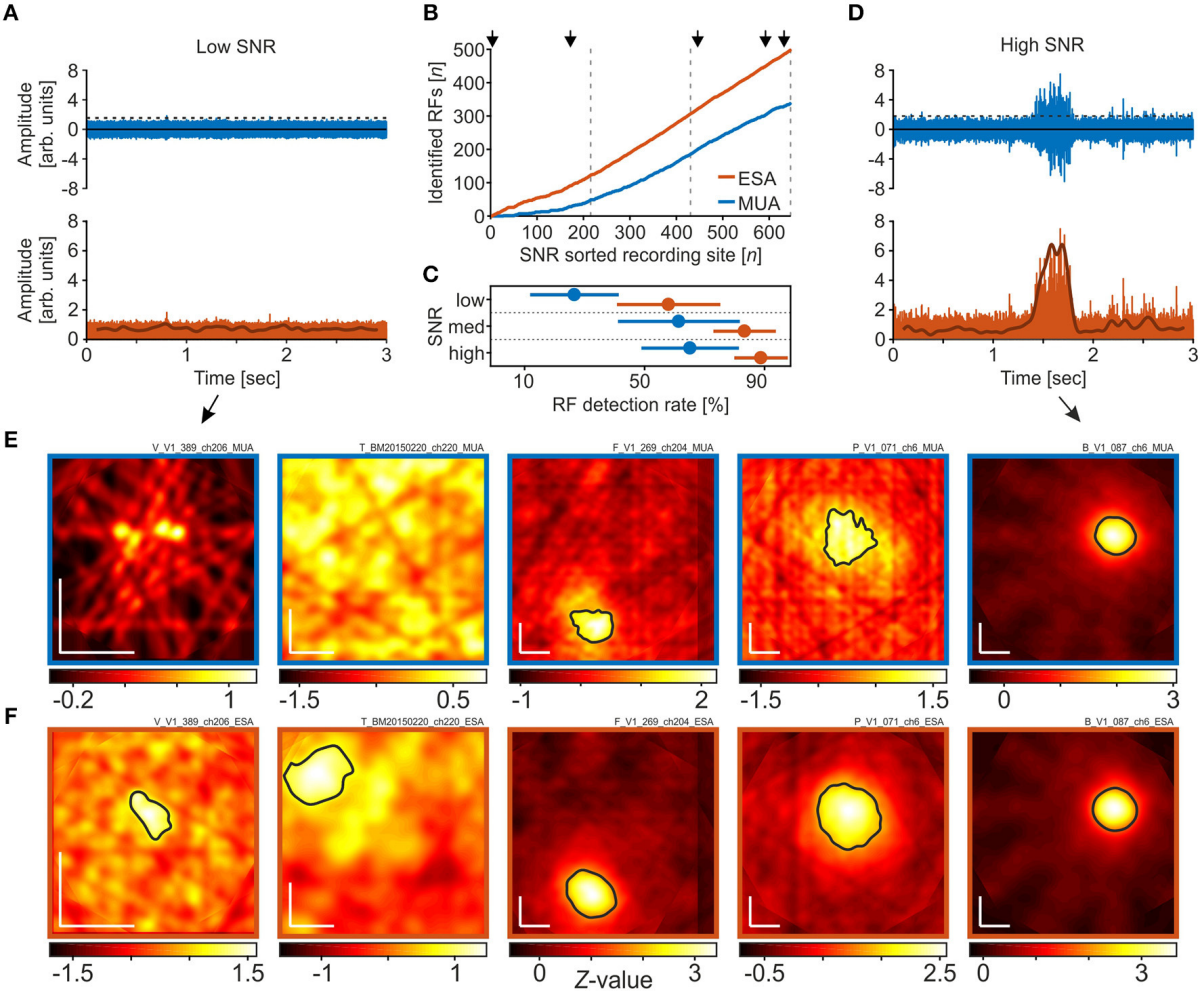
RF detection rates in dependence of signal-to-noise ratio (SNR). **(A)** High-pass filtered neuronal activity with very poor SNR (rank 4 out of 653). The upper panel depicts a single trial in response to a motion direction that reliably modulated the LFP. The dashed line indicates the threshold as calculated over all trials, using the method of Quiroga et al. (2004) with a median multiplication factor of *a* = 2. Events surpassing the threshold are used to calculate the spike density function. The lower panel shows the same high-passed signal after full-wave rectification. Subsequent low-pass filtering provides the ESA-signal (thick line), revealing a small amplitude modulation during the course of the trial. **(B)** Cumulative distribution of the number of RFs detected using either MUA or ESA, sorted by SNR. Dashed lines distinguish equally large fractions of recording sites with low, medium, and high SNR, as used for statistical analysis. Arrows indicate the SNR ranks of the five example sites shown in **(E,F)**. **(C)** Detection rates for low, medium, and high SNR sites, based on the respective detection rates of data from the individual animals, separately for ESA and MUA. Disks and lines indicate mean ± S.D, respectively. **(D)** Same as in **(A)**, for a recording site with high SNR (rank 632). **(E,F)** Visual response maps based on thresholded MUA **(E)** and ESA **(F)** for the five recording sites indicated in **(B)**. SNR rank increases from left to right. Vertical and horizontal white bars in the lower left corners indicate 1° of visual space. Black outlined areas depict significant visual responses (receptive fields). For the two left-most maps in **(E)** no significant visual response was found with any of the thresholds tested during the iterative procedure. Actual maps were calculated based on a median multiplication factor of *a* = 2 for setting the threshold. Remaining maps (middle to right) were calculated after thresholding with *a* = 3.

We next investigated detection rates under different SNR conditions. When recording with (semi-) chronically implanted electrodes or electrode arrays, the electrodes' tips are usually not optimally positioned to the neurons in their vicinity, such that spike amplitudes may surpass background fluctuations only marginally. Figure 3A provides an example of a single trial under such poor SNR conditions. Although there was a significant visual response in the LFP, spike events at this site had very small amplitudes and only a few passed the threshold, calculated based on equation 1 with *a* = 2 (Figure 3A, top panel). The resultant visual response map, computed over all trials, did not reveal any responsive region in the stimulated visual space based on the thresholded MUA (Figure 3E, left panel). Full-wave rectification and low-pass filtering the signal, however, revealed a small amplitude modulation during the course of the trial (Figure 3A, lower panel). Because in the ESA-signal such small modulations can be reliably detected in trials with low SNR, the ESA-based analysis of the same data provided a visual response map with a significant area of activation (Figure 3F, left panel). A second example from a different monkey is presented in the second-most left panels in Figures 3E,F. Under conditions of high SNR, on the other hand, both thresholded MUA and ESA reliably isolate the evoked spiking activity from background noise (Figure 3D), resulting in visual response maps with clearly defined and similar RFs (Figures 3E,F, middle to right panels). However, when based on MUA, the detected RF regions sometimes appear a little bit noisier and smaller (Figures 3E,F, middle and second rightmost panels).

To investigate the relation between RF detection rate and SNR in more detail, we calculated the SNR of all recording sites and determined detection rates as a function of SNR. SNR was calculated by dividing the median amplitude of all spike events surpassing the threshold by the threshold value itself. For reasons of comparability, the threshold was calculated by a multiplication factor of *a* = 3, as applied in the standard procedure. We used the same dataset as before, i.e., we also included those recordings for which we only detected an RF in the LFP to keep the total *N* constant. Figure 3B depicts the cumulative distribution of the number of RFs detected using either ESA or MUA, sorted from low to high SNR. Note that at low SNR, the two traces representing ESA and MUA strongly deviate, and even with medium and high SNR, the ESA slope is still steeper than the MUA slope.

For statistical analysis, we divided the dataset into three equally large groups of sites with low, medium, and high SNR (indicated by dashed lines in Figure 3B), and calculated the RF detection rate of each animal and group. Based on ESA, a significant visual response was detected at 59.3, 84.7, and 90.2% of recording sites with low, medium, and high SNR, respectively. For thresholded MUA, the corresponding detection rates obtained by the standard procedure were 28, 62.8, and 66.5%. A 2-way RM-ANOVA with the factors signal type and SNR revealed a main effect of both factors [signal type: *F*_(1, 2)_ = 14.87, *P* = 0.0182; SNR: *F*_(2, 2)_ = 4.85, *P* = 0.0417, *N* = 5], and no interaction [*F*_(2, 8)_ = 0.018, *P* = 0.548]. *Post-hoc* Tukey HSD multiple comparison tests showed that at low SNR, ESA-based detection rates were higher than MUA-based detection rates at the 95% confidence level, while the difference in detection rates at medium and high SNR was statistically not significant (Figure 3C). The iterative procedure delivered equivalent statistical conclusions.

Additionally, because ESA delivered RFs at recording sites where MUA did not (iterative procedure: *N* = 115), we estimated the likelihood to get a false positive RF detection. This was done by re-shuffling the time bins and labels of the raw PSTH. We then computed visual activity maps as before (cf. section Receptive Field Detection). The actual number of RFs found with this procedure was zero, indicating a very low likelihood that the additionally detected ESA-RFs consist of a significant number of false positives.

### RF Size and Orientation Tuning

**Figure 4:**
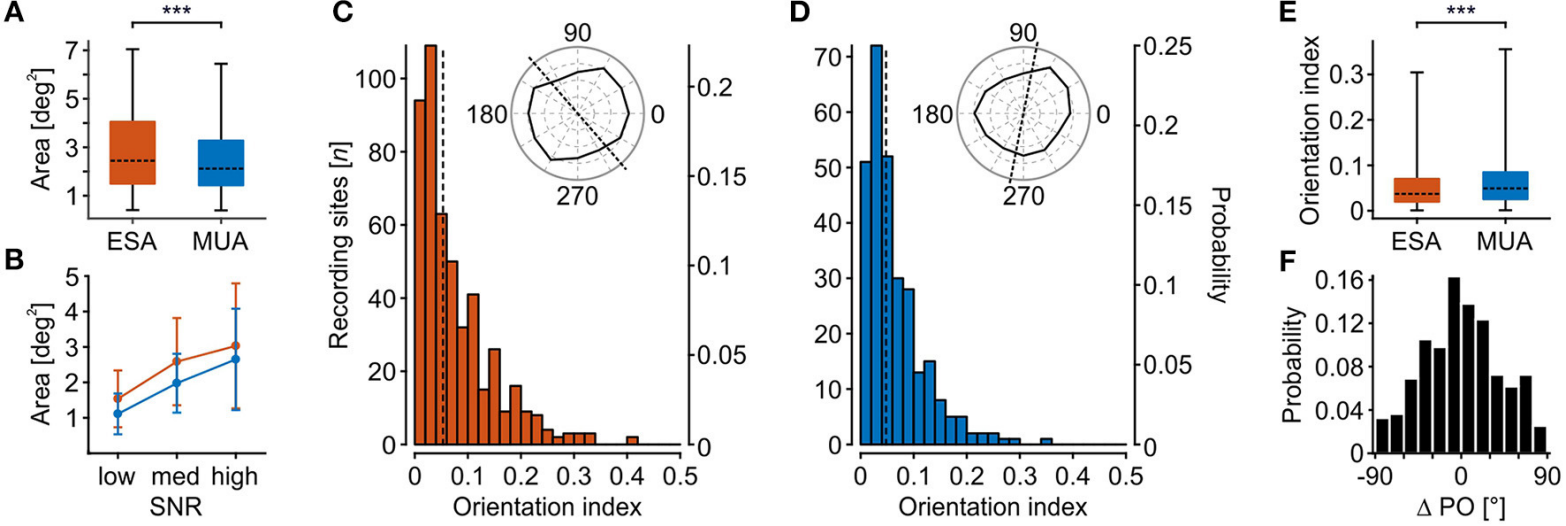
Comparison of stimulus selectivity between ESA and MUA. **(A)** RF area of recording sites with significant visually evoked response modulation for both ESA and MUA (*N* = 385). **(B)** RF area as a function of SNR for same units as in **(A)**. Error bars indicate S.D. **(C,D)** Distribution of orientation indexes for ESA **(C)** and MUA **(D)**. Dotted lines indicate median OI. Polar plot insets show exemplary orientation selective responses at median OI value for either signal type. **(E)** Direct comparison of orientation selectivity of units with significant orientation-selective responses for both MUA and ESA (*N* = 275). **(F)** Signal-dependent difference in preferred orientation (ΔPO) for same units as in **(E)**. Boxplot conventions as in Figure 2. Asterisks indicate statistical difference for α < 0.005% (^***^).

Higher detection rates in data with poor SNR do not necessarily imply that they will provide reliable estimates about the response characteristics of the underlying group of neurons. We therefore investigated the selectivity of ESA and thresholded MUA with respect to the estimated RF size and orientation tuning of the detected units. To get the maximal RF yield, we based this analysis on the iterative procedure for thresholding. Likewise, to obtain the maximal spike information from each unit, we used the smallest threshold that allowed detection of a significant visual response for that unit. Assuming idealized circular RFs, the mean calculated diameter ± S.D. of ESA- and MUA-RFs was 1.6 ± 0.45 and 1.49 ± 0.46°, respectively. For comparison, the size of LFP-RFs was 1.7 ± 0.38° and thus was slightly larger than for ESA and MUA. For statistical analysis, we limited the dataset to those units delivering a significant RF for both ESA and MUA and based the calculation on actual RF areas. ESA-RFs were found to be significantly larger than MUA-RFs (Wilcoxon signed rank test, *Z* = 9.84, *P* < 10^−22^, *N* = 385, *R* = 0.355) (Figure 4A). A Tukey HSD multi-comparison analysis for units with low, medium, and high SNR revealed that the estimated size of both signals increased from low to high SNR (both *P* < 10^−5^, *N* = [30 60 295]), but the size difference between ESA-and MUA-RFs did not differ between SNR groups (1-way ANOVA, *F*_(2, 382)_ = 2.394, *P* = 0.09, *N* = [30 60 295]) (Figure 4B).

Orientation selectivity was estimated based on vector averaging the responses to the six bar orientations, according to Grabska-Barwinska et al. (2012). The method labels orientation tuning as significant depending on the reliability of orientation-related responses over trials, but independent of the absolute orientation index (OI). Orientation tuning was considered significant at 97.8% (*N* = 489) of all recording sites with a significant ESA-RF, and at 72.2% (*N* = 288) of all sites with a significant MUA-RF. Figures 4C,D show the distribution of OI values for the two signal types. Polar plot insets depict two exemplary recording sites with an OI close to the population medians for ESA (0.053) and MUA (0.048).

Statistical analysis was performed on units with a significant orientation tuning for both ESA- and MUA-RFs (Figure 4E). For this subset of sites, MUA turned out to be significantly more orientation selective than ESA (Wilcoxon signed rank test, *Z* = 3.8, *P* < 10^−3^, *N* = 275), but the effect size was small (*R* = 0.162). Despite this reduction in absolute selectivity, 43% (*N* = 117) of the recording sites had about the same preferred orientation (deviation < 15°) independent of signal type, and 70% (*N* = 164) of recording sites did not differ by more than 30°, i.e., preferred orientation was within the range of two neighboring stimulus orientations (Figure 4F).

## Discussion

Full-wave rectification and subsequent low-pass filtering of multi-unit spiking activity was introduced by Eckhorn and colleagues (Eckhorn, 1991, 1992; Eckhorn and Obermüller, 1993; Brosch et al., 1997) to improve spectral frequency analysis of correlated activity, and has been used by several groups as an alternative measure for multi-unit activity (Self et al., 2016; Dougherty et al., 2017; Drebitz et al., 2018). Because this method does not involve setting a threshold for cutting spike amplitudes, full information about the neuronal response is preserved. We hypothesized that this way of pre-processing is particularly effective for data with poor SNR. Even if spike amplitudes do not surpass the background, aggregated spike events in the rectified signal will be reflected in the low-passed derivate due to their different temporal structure, while random background fluctuations get attenuated. Thresholding of such small spikes, on the other hand, is likely to result in both false positive and false negative spike events, thus blurring the available stimulus information.

We tested this hypothesis by using data from semi-chronic recordings of primary visual cortex that was acquired during mapping procedures for testing visually evoked activity. The mapping procedures were performed for different research projects having different target layers within V1. In addition, electrodes were located within the tissue for variable time periods (days to weeks to months), and recording details (hardware, filter) varied across animals. This explains the variance in detection rates across animals (Table 1), but more importantly, it shows that the findings of the current study do not result from specific experimental conditions. Instead, the basically same result across animals suggests a general advantage of ESA over thresholded MUA for detecting evoked activity in the high-frequent signal of neuronal responses. Over all SNR fractions, ESA delivered about 50% more RF estimates than conventional MUA, and was only slightly less sensitive than the broadband-gamma LFP (40–120 Hz). This increased detection rate was mainly due to a much higher sensitivity for detecting RFs in low SNR recordings. With the standard procedure, ESA delivered 2.5 times the number of RFs as compared to MUA. Optimizing the yield of MUA-based detection by iteratively applying a series of thresholds to each unit allowed to increase the number of detected RFs in low SNR data by about 60%, but this was still significantly less than ESA-based detection rates. For medium and high SNR, ESA delivered more RFs than MUA in each individual animal, independent of the procedure, but detection rates for ESA and MUA approximated and were statistically not different over the sample size of five animals.

Average RF size slightly increased from low to high SNR for both ESA and MUA, and ESA-RF size was about 17% larger than MUA-RF size in units delivering an RF with both signals. Yet, this difference was consistent over all SNR fractions, indicating about the same reliability of both signal types. Similarly, ESA-RFs were found to have a slightly smaller absolute orientation selectivity than MUA-RFs, but for 70% of recordings ESA and MUA delivered the same or a very similar preferred orientation. These results support the notion that ESA is a highly sensitive, selective, and reliable signal type significantly increasing the yield of recordings, particularly under conditions that do not allow optimal positioning of electrodes to isolate single units.

### Increased Sensitivity for Detection of Evoked Responses

As a rule of thumb, the amplitude of a spike decays as the inverse of the square of the distance to the recording electrode's tip. For example, the voltage amplitude of a spike generated at a soma with 10–30 μm diameter will decay by about 90% in 60–65 μm distance from the recording electrode tip (Rall, 1962; Lemon, 1984; Gray et al., 1995). Thus, spikes generated at larger distances from the electrode tip get lost in general background noise when not surpassing the threshold, or will be intermixed with noise when threshold is too low. Because of this negative effect on SNR, this introduces a significant limitation for detecting evoked responses. ESA, on the other hand, is sensitive for aggregated spikes even when having small amplitudes, and rather insensitive to random background noise. The resultant signal has a clearly improved SNR, as indicated by the strong increase in the yield of significantly modulated ESA-RFs with low SNR, and even the moderate though insignificant increase in yield for medium and high SNR data.

It is worth to note yet that the division into the three SNR groups is to some extent arbitrary. We divided our dataset into equally large SNR fractions and categorized these as low, medium, and high. Our recordings were obtained from different cortical layers, in many sessions we were primarily interested in the LFP. Thus, only a few data may has been recorded under truly high SNR conditions, while some of the data representing the high SNR pool might has had a weak absolute SNR in fact. Thus, the slightly higher ESA-detection rates for medium and high SNR may disappear under conditions with overall higher SNR. However, our analyses show that ESA is particularly powerful to detect evoked responses when SNR conditions do not allow to set a legitimate threshold. This is particularly evident when comparing ESA detection rates with the optimized yet much weaker detection rates obtained after iteratively searching for the most appropriate threshold of each unit. Such low SNR conditions may result from larger distances between electrode tips and somata when using permanently implanted probes, or from cell loss, gliosis, or local tissue responses potentially associated with (semi-) chronic recording approaches (Turner et al., 1999; Biran et al., 2005; Polikov et al., 2005; Griffith and Humphrey, 2006; Lacour et al., 2016; Salatino et al., 2017), which in turn makes it necessary to exclude single electrodes from further analysis. Here, ESA represents a powerful alternative to conventional thresholding of MUA activity and allows for a strongly increased yield of data, with the additional advantage that its application can be fully automatized.

### Stimulus Selectivity

Because ESA is a neuronal mass signal and reflects the activity of a local population of neurons, the slight differences in RF size and absolute orientation selectivity may primarily be due to a larger group of neurons underlying the ESA-signal as compared to thresholded MUA. Supèr and Roelfsema (2005) compared direction selectivity, response latency, figure ground segregation, and attentional modulation of ESA (denoted as MUA_E_ in their article) to single units. In line with our results, the authors found a somewhat reduced direction selectivity but otherwise largely identical response characteristics. Because axonal and dendritic spikes are very small and the time course of postsynaptic potentials is slow, they concluded that ESA is representing the summed action potentials of neurons with a soma in the vicinity of the recording site rather than electrical fluctuations from other sources. This interpretation also explains the increase in RF size and the reduction of absolute orientation selectivity (Figure 4). Because ESA is not discarding spikes below threshold, it integrates over more sources than conventional MUA, which necessarily results in a somewhat reduced stimulus selectivity. Brosch et al. (1997) specified the effective range of ESA as ~50 μm around the electrode tip. Referring to the classical finding that orientation preference of neurons only 25–50 μm apart from each other may shifts by about 10° (Hubel and Wiesel, 1974), integration of smaller spikes from more distant somata is likely to explain the reduction in absolute orientation selectivity. In addition to this, the higher sensitivity for small spikes prevents, or at least attenuates the typical sampling bias toward large pyramidal neurons when thresholding spikes. Thus, the ESA database may include a larger diversity of cell types than the MUA database, including cells with larger RFs, smaller orientation selectivity, or different center-surround interactions, as found in different layers of V1 (Sceniak et al., 2001; Ringach et al., 2002; Shapley et al., 2003).

Apart from the slightly attenuated total stimulus selectivity, both the analysis of RF size as a function of SNR and cross-comparison of orientation selectivity across signal types primarily revealed that ESA delivers a reliable estimate of the response properties of the recorded group of neurons. First, although RFs were getting slightly larger with better responsiveness of the recording site (due to the reasons outlined above), this increase was found for both signal types and to equal extent. This indicates that even with poor SNR evoked responses were sufficiently well detected to allow for a reasonable estimation of the response properties of the local set of neurons. Second, the estimated preferred orientations were similar between ESA and MUA for the majority of recording sites. Importantly, the method we used for denoting a cell's response as either significantly or insignificantly being influenced by the orientation of the stimulus relies on reproducibility of responses rather than on absolute orientation selectivity. This diminishes the influence of random singular events for estimating response properties of the recorded group of neurons. The finding that almost 98% of the ESA responses were classified as orientation-dependent (as compared to 72% of the MUA responses) proves the high reliability of the ESA-signal to reveal even a small response bias toward one orientation. Detectability of such biases might be important for different purposes, as e.g., for selecting proper stimulus conditions or improving performance of decoding techniques.

Taken together, full-wave rectification and subsequent low-pass filtering of spiking activity effectively increases the signal's SNR and allows for more reliably detecting evoked responses in data with low SNR. Because no thresholding is applied, ESA considers the full spiking information and allows for reliable characterization of the response properties of the underlying group of neurons when conventional techniques may fail.

## Author Contributions

ED, AK, and DW designed the research. ED, BS, and DW performed the research. ED and DW analyzed the data, prepared the figures, and drafted the manuscript. ED, BS, AK, and DW interpreted the results, edited and approved the final version of the manuscript.

### Conflict of Interest Statement

The authors declare that the research was conducted in the absence of any commercial or financial relationships that could be construed as a potential conflict of interest.
